# Risk Factors for Sarcopenia in the Elderly with Type 2 Diabetes Mellitus and the Effect of Metformin

**DOI:** 10.1155/2020/3950404

**Published:** 2020-10-07

**Authors:** Fenqin Chen, Shuai Xu, Yingfang Wang, Feng Chen, Lu Cao, Tingting Liu, Ting Huang, Qian Wei, Guojing Ma, Yuhong Zhao, Difei Wang

**Affiliations:** ^1^Departments of Geriatrics, The First Affiliated Hospital, China Medical University, Shenyang 110001, China; ^2^Department of Clinical Epidemiology, Shengjing Hospital of China Medical University, Shenyang 110004, China; ^3^China Clinical Research Center, Shengjing Hospital of China Medical University, Shenyang, 110004, China; ^4^Department of Geriatrics, Shengjing Hospital of China Medical University, Shenyang 110004, China

## Abstract

**Aims:**

Sarcopenia is a common condition in older individuals, especially in the elderly with type 2 diabetes mellitus (T2DM). The aim of the present study was to examine the risk factors for sarcopenia in elderly individuals with T2DM and the effects of metformin.

**Methods:**

A total of 1732 elderly with T2DM were recruited to this cross-sectional observational study, and we analyzed the data using logistic regression analyses. Skeletal muscle mass, grip strength, and usual gait speed were measured to diagnose sarcopenia according to the criteria of the Asian Working Group for Sarcopenia, combined with expert consensus on sarcopenia in China.

**Results:**

The overall prevalence of sarcopenia was 10.37% of the participants. In the multivariate analysis, sex, age, educational level, and BMI were risk factors for sarcopenia, with women more likely to develop sarcopenia relative to men (OR = 2.539, 95% CI = 1.475–4.371; *P* < 0.05). We observed that sarcopenia increased with age and decreased with increasing BMI and educational level (*P* < 0.05). Participants who took metformin alone or combined with other drugs exhibited a lower risk for sarcopenia than those who took no medication (OR = 0.510, 95% CI = 0.288–0.904 and OR = 0.398, 95% CI = 0.225–0.702, respectively; *P* < 0.05).

**Conclusions:**

We showed that being female and at an older age, lower educational level, and lower BMI were risk factors for sarcopenia in elderly T2DM and that metformin acted as a protective agent against sarcopenia in these patients.

## 1. Introduction

One of the most pressing problems facing mankind today is the aging of the general population, particularly in terms of quality-of-life issues. As a result of population aging, sarcopenia has also become a worldwide social issue [1]. Sarcopenia is a common disorder in elderly populations and is characterized by age-related loss of muscle mass, reduced muscle strength, and/or low physical performance that contributes to functional decline, disability, frailty, and falls [2–4]. In 1989, Rosenberg first introduced the term “sarcopenia” to refer to age-related loss of skeletal muscle mass and volume [5]. Muscle mass accounts for 75% of body cell mass and 45% of body mass [5, 6]; but once individuals reach the age of 50, they tend to lose 1%–2% of their muscle mass per year [2]. The prevalence of sarcopenia as reported in the literature varies considerably, ranging from 1% to 29% in community-dwelling populations to 14%–33% in long-term care populations [7].

China now leads the world in terms of the size of its aging population. Since 1980, the number of people over 60 has grown by an average of 3.2% annually, and currently, the proportion of those 60 years of age and older in China is 17.3% of the total population. In 2017, China became the only country in the world to have more than 200 million elderly [8], and sarcopenia and its associated comorbidities constitute a major health issue for the rapidly aging Chinese population. It was reported that the prevalence of sarcopenia in people aged 60 and older is 10.6% (11.3% in men and 9.8% in women) [9]. In 1069 suburban-dwelling Chinese aged ≥60 years using the Asian Working Group for Sarcopenia (AWGS) definition, the prevalence of sarcopenia was 9.3%—with 6.4% in men and 11.5% in women [10]. In a study population of 887 urban and rural community-dwelling elderly adults aged 60 or older in western China, the prevalence of sarcopenia was 9.8% (women, 12.0%; men, 6.7%) [11].

Diabetes mellitus (DM), a chronic metabolic disease, has reached epidemic status; and it now poses one of the major threats to human health in the 21st century. In 2017, the International Diabetes Federation (IDF) estimated that 425 million individuals worldwide were suffering from DM, and it is expected that the number will rise to 629 million by 2045 [12]. Approximately 90% of these individuals have type 2 diabetes mellitus (T2DM) [13], with the highest prevalence observed in older adults [14]. Because of advancements in the management of DM and DM comorbidities, life expectancy in DM patients has been extended, and this has led to an increase in the number of older individuals with DM. More importantly, it has recently been reported that elderly patients with diabetes are at an increased risk for sarcopenia [15]. Diabetes, which is associated with reduced muscle strength and poor muscle quality, may be considered an “accelerated aging process” that intensifies age-related sarcopenia [16]. Sarcopenia then impairs activities of daily living and increases the risk of mortality in elderly adults with diabetes [17, 18]. Although the underlying reason for DM often coexisting with sarcopenia has not been fully elucidated, several lines of evidence have shown that some mechanisms subserving sarcopenia are closely associated with DM pathophysiology [17]. As diabetes is a known risk factor for sarcopenia, screening and diagnosis in diabetic older individuals appear to be a step in the right direction.

Metformin lowers blood glucose levels by sensitizing the liver to the effects of insulin, thus suppressing hepatic glucose output. According to updated guidelines, metformin is considered a first-line treatment, including treatment of the elderly [19]. It has been reported that metformin exerts positive effects on muscle mass and strength [20–23], although other studies have suggested that metformin was ineffective [24–27]. Whether metformin exerts positive or negative effects with respect to sarcopenia is unknown, and only a few studies have focused on the risk factors for sarcopenia in elderly T2DM patients. Therefore, the aims of the present study were to estimate the prevalence and risk factors associated with sarcopenia in elderly T2DM in China and also to investigate the effects of metformin on sarcopenia in this specific population. This trial was registered at the China Clinical Trial Center (ChiCTR-ERC-17011100).

## 2. Methods

### 2.1. Study Design and Participants

**Figure 1:**
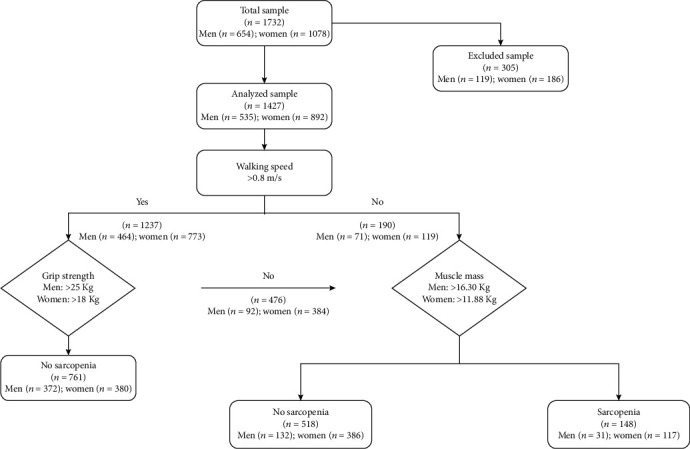
Flow chart of sarcopenia screening and assessment

We used the United Nations (UN) guideline of 60+ years to define the elderly population [28], and the source population consisted of nine community residences in Shenyang, Liaoning, which is located in northeastern China. The inclusion criteria for this cross-sectional study were patients over 60 years of age with type 2 diabetes and those who were physically active and agreed to participate. The participant groups were treated with either diet alone (control), oral hypoglycemic agents alone, or a combination of both oral hypoglycemic agents and insulin injections. Participants were excluded from the study if they exhibited the following: (i) a history of stroke, (ii) carpal tunnel syndrome, (iii) severe hip or knee osteoarthritis, (iv) inability to perform the handgrip strength test or 4-meter walking test, or (v) if they lacked complete data. As no one in our study used thiazolidinedione or sodium-glucose cotransporter 2 (SGLT2) inhibitors and fewer than 10 people used glucagon-like peptide 1 receptor agonist (GLP-1) inhibitors and dipeptidyl peptidase 4 (DPP4) inhibitors, we excluded them for statistical purposes. Finally, 1427 patients with type 2 diabetes were selected from the overall population between May 2017 and August 2019 (Figure 1). All of the participants provided written consent after being informed regarding the use of their personal information and of their benefits, medical programs, and confidentiality agreements. For illiterate participants, we obtained written informed consent from their relatives. All of the procedures were conducted in accordance with ethical standards. The study was approved by the Ethics Committee of China Medical University (Shenyang, China, AF-SOP-07-1. 0-01).

### 2.2. Study Consent and Measurement

Data were collected during a single visit to the clinic by endocrinologists and trained nurses from a face-to-face interview that entailed a standard questionnaire, as reported previously [8]. Data on demographic characteristics, such as name, sex, age, educational level attained, and marriage status; previous medical history, including hypertension, diabetes, kidney disease, or other health problems; and lifestyle factors, such as smoking, drinking, and diet, were obtained by interview with a standardized questionnaire. The total cohort was divided into a nonsmoking group, a smoking group, and a smoking-cessation group with respect to smoking status. Alcohol imbibition over the previous year was based upon drinking at least twice a week, with an average alcohol consumption of 40 g during one drinking session. Patients were then divided into a nondrinking group, a drinking group, and a drinking-cessation group. Educational level was divided into primary school or below, middle school, and high school or above. Exercise was divided into regular exercise, occasional exercise, and no exercise.

Physical examinations included weight, height, waist circumference, hip circumference, and blood pressure. Body weight was measured using digital scales to the nearest 0.1 kg, and height was assessed to the nearest 0.1 cm using a wall-mounted stadiometer; both measurements were procured while the participants were fasting and wearing light clothes and were without socks or shoes. We then calculated the body mass index (BMI, kg/m^2^). Waist circumference (WC) was measured as follows. The feet were separated by 30–40 cm (approximately the width of the shoulders), and a soft ruler was used to measure the circumference of the body at the midpoint of the connecting line between the anterior superior iliac crest and the 12th costal margin. Hip circumference (HC) was measured around the widest portion of the pelvis. Both WC and HC were determined using the same measuring tape, and the soft ruler was applied to the skin without compression. We use a standardized automatic electronic sphygmomanometer (HEM7200; Omron Healthcare, Kyoto, Japan) to measure blood pressure (BP) three times at 3 min intervals followed by at least 5 min of rest. The mean of the three BP measurements was then calculated and used for all analyses. The participants were seated with their arms supported at the level of their hearts throughout these measurements. Blood samples were taken before breakfast in the morning and after an 8–12-hour overnight fast. Fasting blood glucose (FBG), glycosylated hemoglobin (HbA1c), total cholesterol (TC), triglycerides (TG), low-density lipoprotein cholesterol (LDL-C) and high-density lipoprotein cholesterol (HDL-C), total protein (TP), albumin (Alb), and hemoglobin (HB) were measured in all of the subjects.

### 2.3. Definition of Sarcopenia

#### 2.3.1. Measurement of Skeletal Muscle Mass

Dual-energy X-ray absorptiometry (DEXA), magnetic resonance imaging (MRI), computed tomography (CT), and bioimpedance analysis (BIA) are recommended by AWGS for measuring muscle mass. However, these sophisticated medical instruments are rarely available in communities of northeastern China, and they are too expensive to be used for screening sarcopenia. Therefore, we decided to apply weight and body fat measures for estimating muscle mass because of the low cost and ease of application. We used a V. Body HBF-701 (Omron Healthcare, Kyoto, Japan)—which is also a muscle rate analyzer—for the evaluation of body composition, we obtained the data for body weight (kg), body fat mass rate (%), body muscle rate (%), and appendicular muscle mass (%). Muscle mass was defined as the weight multiplied by the whole-body muscle rate. To calculate the cutoff values for muscle mass, we selected a reference group of young participants aged 18–40 years who had completed the measures (141 males and 150 females). Low muscle mass was defined as muscle mass values less than double the standard deviation of the normal population. In our study, the cutoff value used to define low muscle mass was 16.30 kg for males and 11.88 kg for females.

#### 2.3.2. Measurement of Muscle Strength (Handgrip Strength)

Handgrip strength (HS) was measured to the nearest 0.1 kg using a handheld dynamometer based on strain-gauge sensors (WCS-100; Yilian Inc., Shanghai, China). The measurement for each subject was performed on both arms and repeated three times, and the highest value for either hand was then used for the analyses [29]. Combined with the recommendations of the AWGS [3] and expert consensus on sarcopenia in China [30], a low HS was defined as <25 kg for men and <18 kg for women.

#### 2.3.3. Measurement of Physical Performance

According to the AWGS2014 [3] and expert consensus on sarcopenia in China [30], physical performance was assessed using usual gait speed (GS). In order to measure usual GS, the participants were asked to walk a 20 m course at their usual speed. To perform this, the subjects were required to start their foot movements when the timing commenced and stop when one foot contacted the ground after completely crossing the 20 m mark. Low physical performance was defined as a usual GS of less than 0.8 m/s.

#### 2.3.4. Diagnosis of Sarcopenia

As our study and data processing commenced prior to AWGS2019 [4], our diagnosis was still in accordance with the standards set by the AWGS2014 [3], combined with expert consensus on sarcopenia in China [30], and it was defined as a presentation of both low muscle mass and function (strength or performance). Low physical performance was defined as gait speed < 0.8 m/s, and low muscle strength was defined as a handgrip strength < 25 kg in men and <18 kg in women. Low muscle mass was defined when the muscle mass values were less than double the standard deviation for the normal population. For this study, we defined the cutoff value for low muscle mass as 16.30 kg in men and 11.88 kg in women.

## 3. Statistical Analysis

Epidate was used to build the database, and we performed statistical analyses on the sarcopenic and nonsarcopenic groups. We used multiple imputation—based on the Markov chain Monte Carlo method in the SAS MI procedure—to account for missing data. Continuous variables are presented as means ± 1 standard deviation (SD), and categorical are presented as frequencies (%). The statistical significance of the differences between groups was assessed using a chi-squared test for categorical variables and a two-sample *t*-test for continuous variables. Multivariate logistic regression analyses were used to calculate the odds ratios (ORs) and 95% confidence intervals (CIs) for the effects of several factors on the presence of sarcopenia. Our statistical analyses were performed using SAS Version 9.4 (SAS Institute, Cary, NC) and two-sided tests. A *P* value < 0.05 was considered to be significant.

## 4. Results

In the present study, a total of 1732 participants with T2DM were screened: 654 men and 1078 women. Of the total, 305 participants were excluded for either incomplete data or for using GLP-1 agonists and/or DPP4 inhibitors. We thus ultimately analyzed 1427 participants, of which 535 were men and 892 were women (Figure 1).

The clinical characteristics of our study population are summarized in Table 1, with sarcopenia present in 10.37% (148/1427) of the participants. A chi-squared test showed that the prevalence of sarcopenia varied greatly by sex (*P* < 0.001): in men, it was 5.79%, while in women, it was 13.11%. When the prevalence of sarcopenia was evaluated by age group, it significantly increased with age—from 6.51% in the 60- to 69-year-old group to 27.67% for those >80 years of age (*P* < 0.001). As BMI and educational level increased, the incidence of sarcopenia decreased commensurately (*P* < 0.001). Although sarcopenia was associated with alcohol intake, we showed that its incidence was relatively low in persons with alcohol intake. With the increase in TC, the prevalence of sarcopenia increased, whereas the relationship between TG and sarcopenia was inversely proportional. As for hypoglycemic drug use, the prevalence of sarcopenia among those who did not take any hypoglycemic drugs was 17.12%, whereas the prevalence of the metformin-only group and the metformin combined group was 8.1% and 6.67%, respectively (*P* < 0.001). There were no significant differences between nonsarcopenic and sarcopenic groups when further subdivided for smoking, exercise, HB, presence of hypertension, TP, Alb, HDL-C, LDL-C, FBG, or HbA1c (*P* > 0.05, Table 1).

Table 2 shows the factors involved in the diagnosis of sarcopenia. Grip strength, usual gait speed, and muscle mass were lower in the sarcopenic group relative to the nonsarcopenic group in both men and women (*P* < 0.001), except for usual gait speed in men (*P* < 0.05).

The results of the multivariate logistic regression analyses are detailed in Table 3. Being female and older age were obvious risk factors for sarcopenia, whereas education, BMI, and hypoglycemic drugs (both metformin alone or combined with other drugs) were protective factors for sarcopenia. Participants taking metformin alone or with other drugs exhibited a lower risk of sarcopenia compared to those taking no medication (adjusted OR = 0.510, 95% CI = 0.288–0.904; OR = 0.398, 95% CI = 0.225–0.702, respectively; *P* < 0.05), whereas alcohol use, TC, and TG were not associated with the presence of sarcopenia.

## 5. Discussion

To the best of our knowledge, this is the first study aimed at detecting the risk factors for sarcopenia in elderly T2DM patients and also at detecting the effects of metformin on the condition according to the AWGS criteria, combined with expert consensus on sarcopenia in China.

There are notable differences in the reported prevalence of sarcopenia, which may be affected by the study and reference populations [31], and even when the AWGS criteria are used, the prevalence still varies. A study from Japan suggested that the prevalence of sarcopenia among community-dwelling older adults aged 65–89 years was 21.8% in men and 22.1% in women [32], while another Japanese study suggested that it was 9.6% and 7.7%, respectively [33]. In China, the prevalence of sarcopenia in individuals aged 60 and over is 10.6% (11.3% in men and 9.8% in women) [9], and its prevalence among elderly men and women in Taiwan was 9.3% and 4.1%, respectively [34]. Investigators from Hong Kong showed that the prevalence of sarcopenia among elderly male community dwellers was 9.4% [35]. Other research has also shown that older adults with T2DM have an accelerated loss in muscle mass and strength compared with adults without diabetes [18, 36] and that the prevalence of sarcopenia in patients with diabetes was significantly higher than in nondiabetic subjects. A Singapore study showed that the prevalence of sarcopenia in older, community-dwelling patients with T2DM was 27.4% [37], and another from China depicted a significantly higher prevalence of sarcopenia in T2DM patients relative to healthy controls (14.8% vs. 11.2%, *P* = 0.035) [29]. From our study, we concluded that sarcopenia was present in 10.37% of all of the participants, with 5.79% in men and 13.11% in women. The prevalence of sarcopenia in our study was relatively lower than in the aforementioned study—primarily reflected in the prevalence in men—which may be related to the criteria we adopted for the diagnosis of sarcopenia. Although our study was also based on AWGS criteria, we combined those with expert consensus on sarcopenia in China, and our diagnosis of handgrip strength < 25 kg in men—which was different from the AWGS criterion of <26 kg—may have led to a lower prevalence of sarcopenia in males.

The prevalence of sarcopenia increases with age [31], and our research also reflected this characteristic. The prevalence of sarcopenia in individuals who are 60–69 years of age was 6.51%, 13.63% for those 70–79, and 27.67% for those over 80—which was consistent with several other studies [10, 37, 38].

Sarcopenia is also related to sex of the individual, but its impact on the prevalence of sarcopenia differs among studies. Some studies showed a higher prevalence among men [9, 34, 39], whereas others depicted a higher prevalence among women [10, 11]. We concluded from our work that the prevalence for women was higher than for men (13.11% vs. 5.79%, respectively), which may have been due to the different diagnostic criteria used for men and women. Another reason may be the nature of Chinese culture. In China, men usually go out for shopping and do other outside activities, while women do more housework at home. Men are relatively more active than women.

Although the prevalence of obesity in combination with sarcopenia is increasing in older adults [40], the authors of one study have suggested that the greater muscle mass of overweight and obese older adults does not confer a functional advantage [41] although other studies have shown that BMI was found to be negatively associated with sarcopenia [38, 42]. We also concluded that BMI was negatively correlated with sarcopenia; that is, the prevalence of sarcopenia decreased as BMI increased. This suggested to us that weight may exert a protective effect against sarcopenia.

We also studied the relationship between educational level and sarcopenia and found that the higher the educational level, the lower the prevalence of sarcopenia. This is consistent with some studies [43, 44]. However, some other investigations disagreed with this conclusion [39, 45], possibly because the composition of their study participants differed from ours. Also, another reason may be that the higher the education level, the relatively higher the income. This ensures a reasonable and healthy diet. At the same time, these people have high health awareness and can do regular physical exercises. Alcohol has been reported to be another risk factor for sarcopenia [10], but we observed no correlation between alcohol and smoking with sarcopenia, and this was consistent with the conclusions of a previous study [42]. Regarding the relationships between blood glucose or blood pressure and sarcopenia, Mesinovic et al. asserted that any associations between augmented fasting glucose and components of sarcopenia were nonsignificant, as was the association between high blood pressure and sarcopenia [41]. Another study showed that the incidence of sarcopenia in both men and women was not associated with HbA1c [38]. Collectively, these findings were consistent with ours, as we concluded that the prevalence of sarcopenia was not associated with FBG, HbA1c, or blood pressure. Although the decline in motor ability associated with age was the principal factor related to sarcopenia in the elderly [46], we did not find a correlation between exercise and sarcopenia—and this may have to do with the fact that our study only included individuals who were able to walk without assistance.

Metformin, as a first-line hypoglycemic drug, also plays an important role in improving sarcopenia [20–23, 47]. A study from Iran showed that of 51 individuals newly diagnosed with T2DM and placed on 1000 mg of metformin twice daily for six months, the drug postponed the appearance of sarcopenia—especially in women with T2DM who were at higher risk for the loss of skeletal muscle mass [47]. Another study showed that subjects with risk factors for T2DM who received 850 mg of metformin twice a day for two months had a diminution in fat weight and an elevation in lean weight [21]. In the Osteoporotic Fractures in Men (MrOS) study, 151 diabetics were treated with insulin sensitizers, and 111 diabetics were treated without insulin sensitizers over 3.5 ± 0.7 years of follow-up. The study revealed that diabetic men on metformin or metformin coupled with thiazolidinediones demonstrated a significantly attenuated loss of total or appendicular lean mass relative to men with untreated diabetes or diabetes treated without insulin sensitizers [22]. Other studies, however, suggested that metformin might negatively impact mitochondrial function in skeletal muscle [26, 27]. In our study, we found that in the elderly with T2DM, those on metformin (regardless of whether it was metformin alone or combined with other oral hypoglycemic drugs and insulin) manifested a reduced risk for sarcopenia relative to those patients on no medication. However, we did not find any differences in our endpoints between metformin and other hypoglycemic drugs.

Several strengths and limitations should be taken into account when considering the results of this study. This study has a number of strengths. We are the first to establish the risk factors for sarcopenia in elderly patients with T2DM and also the first to evaluate the effects of metformin on this condition—combining the AWGS criteria with expert consensus on sarcopenia in China. Second, this study was conducted on a relatively large sample of well-characterized, urban-dwelling elderly men and women living in a defined geographic area. Third, both sarcopenic and nonsarcopenic patients were recruited by the same research assistant from the same population and from the same source to reduce confounding bias. Fourth, the diagnostic guidelines that we used for sarcopenia were according to the AWGS criteria in combination with expert consensus on sarcopenia in China, which are more suitable for the Chinese population. Despite extensive efforts to curb them, there were also a number of limitations to our study. First, this study used a cross-sectional design, so it was not possible to determine causal relationships. Second, all of the participants in the present study were physically active; we did not include participants who demonstrated a history of stroke, carpal tunnel syndrome, and severe hip or knee osteoarthritis or were unable to perform a handgrip strength test or the 4-meter walking test. Because of this, our results might in fact underestimate the prevalence of sarcopenia. Third, although DEXA, magnetic resonance imaging, computed tomography, or BIA are recommended for measuring muscle mass by the AWGS, we used a muscle-rate analyzer for the evaluation of body composition. Fourth, although one aim of our study was to investigate the effects of metformin on sarcopenia, other hypoglycemic drugs in our study included sulfonylureas/glinides, alpha-glucosidase inhibitors, and insulin, and these were not specifically subcategorized, even though they might also have elicited protective effects against sarcopenia.

In summary, we examined the prevalence of sarcopenia and determined several associated factors in an elderly population with T2DM. We also assessed the effect of metformin on the sarcopenic condition using the AWGS definition, combined with expert consensus on sarcopenia in China. We found that being female, older age, lower educational level, and lower BMI were risk factors for sarcopenia in elderly diabetic patients and that metformin acted as a protective factor against sarcopenia in our patients. Our study also showed that other hypoglycemic drugs (sulfonylureas/glinides, alpha-glucosidase inhibitors, and insulin) might exert a protective effect on sarcopenia.

## Figures and Tables

**Table 1:** Univariate analysis of baseline characteristics in elderly sarcopenia vs. nonsarcopenia subjects.

Parameter	Total (*N* = 1427)	Nonsarcopenia (*N* = 1279)	Sarcopenia (*N* = 148)	*P* value
Gender				<0.0001
Men	535 (37.5)	504 (39.4)	31 (20.9)	
Women	892 (62.5)	775 (60.6)	117 (79.1)	
Age				<0.0001
60-69	875 (61.3)	818 (64.0)	57 (38.5)	
70-79	440 (30.8)	380 (29.7)	60 (40.5)	
80-	112 (7.8)	81 (6.3)	31 (20.9)	
Education				<0.0001
Primary and below	204 (14.3)	155 (12.1)	49 (33.1)	
Middle school	994 (69.7)	912 (71.3)	82 (55.4)	
College and above	229 (16.0)	212 (16.6)	17 (11.5)	
BMI				<0.0001
<24	562 (39.4)	446 (34.9)	116 (78.4)	
24~28	634 (44.4)	608 (47.5)	26 (17.6)	
≥28	231 (16.2)	225 (17.6)	6 (4.1)	
Smoking				0.1272
Never	1158 (81.1)	1029 (80.5)	129 (87.2)	
Current	213 (14.9)	197 (15.4)	16 (10.8)	
Former	56 (3.9)	53 (4.1)	3 (2.0)	
Alcohol use				0.0236
Never	1088 (76.2)	962 (75.2)	126 (85.1)	
Current	319 (22.4)	299 (23.4)	20 (13.5)	
Former	20 (1.4)	18 (1.4)	2 (1.4)	
Exercise				0.8551
Regular exercise	1093 (76.6)	980 (76.6)	113 (76.4)	
Occasional exercise	47 (3.3)	41 (3.2)	6 (4.1)	
No exercise	287 (20.1)	258 (20.2)	29 (19.6)	
Hypertension				0.2715
No	803 (56.3)	726 (56.8)	77 (52.0)	
Yes	624 (43.7)	553 (43.2)	71 (48.0)	
HB				0.197
<120 (110 female)	43 (3.0)	36 (2.8)	7 (4.7)	
≥120 (110 female)	1384 (97.0)	1243 (97.2)	141 (95.3)	
TP				0.0642^∗^
<66	29 (2.0)	29 (2.3)	0(0)	
≥66	1398 (98.0)	1250 (97.7)	148 (100)	
Alb				0.4957^∗∗^
<38	4 (0.3)	4 (0.3)	0(0)	
≥38	1423 (99.7)	1275 (99.7)	148 (100)	
TC				0.0255
<4.14	282 (19.8)	263 (20.6)	19 (12.8)	
≥4.14	1145 (80.2)	1016 (79.4)	129 (87.2)	
TG				0.0027
<1.7	799 (56.0)	699 (54.7)	100 (67.6)	
≥1.7	628 (44.0)	580 (45.3)	48 (32.4)	
HDL-C				0.3102
<0.9 (1.0 female)	161 (11.3)	148 (11.6)	13 (8.8)	
≥0.9 (1.0 female)	1266 (88.7)	1131 (88.4)	135 (91.2)	
LDL-C				0.9296
<2.6	439 (30.8)	393 (30.7)	46 (31.1)	
≥2.6	988 (69.2)	886 (69.3)	102 (68.9)	
FBG				0.5597
<7	606 (42.5)	547 (42.8)	59 (39.9)	
7~11.1	689 (48.3)	617 (48.2)	72 (48.6)	
≥11.1	132 (9.3)	115 (9.0)	17 (11.5)	
HbA1c				0.6718
<7	822 (57.6)	741 (57.9)	81 (54.7)	
7~9	446 (31.3)	395 (30.9)	51 (34.5)	
≥9	159 (11.1)	143 (11.2)	16 (10.8)	
Hypoglycemic drugs				<0.0001
No medication	333 (23.3)	276 (21.6)	57 (38.5)	
Non-metformin	480 (33.6)	434 (33.9)	46 (31.1)	
Metformin only	284 (19.9)	261 (20.4)	23 (15.5)	
Combination of metformin and other drugs	330 (23.1)	308 (24.1)	22 (14.9)	

^∗^Fisher's exact test. ^∗∗^Continuity Adj. chi-square. BMI: body mass index; HB: hemoglobin; TP: total protein; Alb: albumin; TC: total cholesterol; TG: triglyceride; HDL-C: high-density lipoprotein cholesterol; LDL-C: low-density lipoprotein cholesterol; FBG: fasting blood glucose; HbA1c: glycated hemoglobin.

**Table 2:** Factors for diagnosis of sarcopenia.

Variables	Total	Nonsarcopenia	Sarcopenia	*P* value
Grip strength				
Men	30.9 ± 8.0	31.4 ± 7.8	22.2 ± 5.5	<0.0001
Women	17.8 ± 5.5	18.5 ± 5.3	12.8 ± 3.9	<0.0001
Usual gait speed				
Men	1.1 ± 0.3	1.1 ± 0.3	0.9 ± 0.4	0.0147
Women	1.1 ± 0.3	1.1 ± 0.3	1.0 ± 0.3	<0.0001
Muscle mass				
Men	20.4 ± 4.2	20.8 ± 4.1	14.5 ± 1.7	<0.0001
Women	14.6 ± 3.4	15.1 ± 3.3	10.7 ± 1.0	<0.0001

Data are presented as mean ± standard deviation. Comparison of nonsarcopenia and sarcopenia. SMI: skeletal muscle mass index.

**Table 3:** The results of multivariate analysis of influencing factors of sarcopenia in subjects.

Variables	Reference	OR	95% CI	*P* value
Gender				
Women	Men	2.539	1.475~4.371	0.0008
Age				
70-79	60-69	2.186	1.420~3.365	0.0004
80-		5.191	2.792~9.651	<0.0001
Education				
Middle school	Primary and below	0.408	0.249~0.669	0.0004
College and above		0.360	0.182~0.711	0.0033
BMI				
24-28	<24	0.145	0.090~0.233	<0.0001
≥28		0.075	0.031~0.181	<0.0001
Alcohol use				
Current	Never	1.088	0.592~2.001	0.7850
Former		1.078	0.198~5.863	0.9311
TC				
≥4.14	<4.14	1.657	0.949~2.892	0.0759
TG				
≥1.7	<1.7	0.672	0.442~1.020	0.0620
Hypoglycemic drugs				
Non-metformin	No medication	0.452	0.283~0.721	0.0009
Metformin only		0.510	0.288~0.904	0.0211
Combination of metformin and other drugs		0.398	0.225~0.702	0.0015

BMI: body mass index; TC: total cholesterol; TG: triglyceride; CI: confidence interval.

## Data Availability

All data generated or analyzed during this study are included in the manuscript. The datasets used and/or analyzed during the current study are available from the corresponding author (dfwang@cmu.edu.cn) upon reasonable request.

## References

[1] Fielding R. A., Vellas B., Evans W. J. (2011). Sarcopenia: an undiagnosed condition in older adults. Current consensus definition: prevalence, etiology, and consequences. International working group on sarcopenia. *Journal of the American Medical Directors Association*.

[2] Wang C., Bai L. (2012). Sarcopenia in the elderly: basic and clinical issues. *Geriatrics & Gerontology International*.

[3] Chen L.-K., Liu L.-K., Woo J. (2014). Sarcopenia in Asia: consensus report of the Asian Working Group for Sarcopenia. *Journal of the American Medical Directors Association*.

[4] Chen L.-K., Woo J., Assantachai P. (2020). Asian Working Group for Sarcopenia: 2019 consensus update on sarcopenia diagnosis and treatment. *Journal of the American Medical Directors Association*.

[5] Hong H. C., Hwang S. Y., Choi H. Y. (2014). Relationship between sarcopenia and nonalcoholic fatty liver disease: the Korean Sarcopenic Obesity Study. *Hepatology*.

[6] Nair K. S. (2000). Age-related changes in muscle. *Mayo Clinic Proceedings: 2000*.

[7] Cruz-Jentoft A. J., Landi F., Schneider S. M. (2014). Prevalence of and interventions for sarcopenia in ageing adults: a systematic review. Report of the International Sarcopenia Initiative (EWGSOP and IWGS). *Age and Ageing*.

[8] Chen F., Wei G., Wang Y. (2019). Risk factors for depression in elderly diabetic patients and the effect of metformin on the condition. *BMC Public Health*.

[9] Hai S., Wang H., Cao L. (2017). Association between sarcopenia with lifestyle and family function among community-dwelling Chinese aged 60 years and older. *BMC Geriatrics*.

[10] Han P., Kang L., Guo Q. (2015). Prevalence and factors associated with sarcopenia in suburb-dwelling older Chinese using the Asian Working Group for Sarcopenia definition. *Journals of Gerontology Series A: Biomedical Sciences and Medical Sciences*.

[11] Gao L., Jiang J., Yang M., Hao Q., Luo L., Dong B. (2015). Prevalence of sarcopenia and associated factors in Chinese community-dwelling elderly: comparison between rural and urban areas. *Journal of the American Medical Directors Association*.

[12] Ogurtsova K., da Rocha F. J., Huang Y. (2017). IDF diabetes atlas: global estimates for the prevalence of diabetes for 2015 and 2040. *Diabetes Research and Clinical Practice*.

[13] Association AD (2012). Diagnosis and classification of diabetes mellitus. *Diabetes care*.

[14] Kautzky-Willer A., Harreiter J., Pacini G. (2016). Sex and gender differences in risk, pathophysiology and complications of type 2 diabetes mellitus. *Endocrine Reviews*.

[15] Park S. W., Goodpaster B. H., Strotmeyer E. S. (2006). Decreased muscle strength and quality in older adults with type 2 diabetes: the health, aging, and body composition study. *Diabetes*.

[16] Leenders M., Verdijk L. B., van der Hoeven L. (2013). Patients with type 2 diabetes show a greater decline in muscle mass, muscle strength, and functional capacity with aging. *Journal of the American Medical Directors Association*.

[17] Kalyani R. R., Corriere M., Ferrucci L. (2014). Age-related and disease-related muscle loss: the effect of diabetes, obesity, and other diseases. *The lancet Diabetes & endocrinology*.

[18] Park S. W., Goodpaster B. H., Lee J. S. (2009). Excessive loss of skeletal muscle mass in older adults with type 2 diabetes. *Diabetes Care*.

[19] Association AD (2017). 6. Glycemic targets: standards of medical care in diabetes—2018. *Diabetes care*.

[20] Long D. E., Peck B. D., Martz J. L. (2017). Metformin to Augment Strength Training Effective Response in Seniors (MASTERS): study protocol for a randomized controlled trial. *Trials*.

[21] Rodríguez-Moctezuma J., Robles-López G., López-Carmona J., Gutiérrez-Rosas M. (2005). Effects of metformin on the body composition in subjects with risk factors for type 2 diabetes. *Diabetes, Obesity and Metabolism*.

[22] Lee C. G., Boyko E. J., Barrett-Connor E. (2011). Insulin sensitizers may attenuate lean mass loss in older men with diabetes. *Diabetes Care*.

[23] Pavlidou T., Marinkovic M., Rosina M. (2019). Metformin delays satellite cell activation and maintains quiescence. *Stem Cells International*.

[24] Dungan C. M., Li Z., Wright D. C., Williamson D. L. (2016). Hyperactive mTORC1 signaling is unaffected by metformin treatment in aged skeletal muscle. *Muscle & Nerve*.

[25] Mennes E., Dungan C. M., Frendo-Cumbo S., Williamson D. L., Wright D. C. (2013). Aging-associated reductions in lipolytic and mitochondrial proteins in mouse adipose tissue are not rescued by metformin treatment. *Journals of Gerontology Series A: Biomedical Sciences and Medical Sciences*.

[26] Wessels B., Ciapaite J., van den Broek N. M., Nicolay K., Prompers J. J. (2014). Metformin impairs mitochondrial function in skeletal muscle of both lean and diabetic rats in a dose-dependent manner. *PLoS One*.

[27] Kane D. A., Anderson E. J., Price J. W. (2010). Metformin selectively attenuates mitochondrial H2O2 emission without affecting respiratory capacity in skeletal muscle of obese rats. *Free Radical Biology and Medicine*.

[28] Organization WH (2014). *Definition of an older or elderly person*.

[29] Wang T., Feng X., Zhou J. (2016). Type 2 diabetes mellitus is associated with increased risks of sarcopenia and pre-sarcopenia in Chinese elderly. *Scientific Reports*.

[30] Chinese Society of Osteoporosis and Bone Mineral Research (2016). Consensus on sarcopenia. *Chinese Journal of Osteoporosis and Bone Mineral Research*.

[31] Kim H., Hirano H., Edahiro A. (2016). Sarcopenia: prevalence and associated factors based on different suggested definitions in community-dwelling older adults. *Geriatrics & Gerontology International*.

[32] Yamada M., Nishiguchi S., Fukutani N. (2013). Prevalence of sarcopenia in community-dwelling Japanese older adults. *Journal of the American Medical Directors Association*.

[33] Yuki A., Ando F., Otsuka R., Matsui Y., Harada A., Shimokata H. (2015). Epidemiology of sarcopenia in elderly Japanese. *The Journal of Physical Fitness and Sports Medicine*.

[34] Huang C.-Y., Hwang A.-C., Liu L.-K. (2016). Association of dynapenia, sarcopenia, and cognitive impairment among community-dwelling older Taiwanese. *Rejuvenation Research*.

[35] Yu R., Leung J., Woo J. (2014). Incremental predictive value of sarcopenia for incident fracture in an elderly Chinese cohort: results from the Osteoporotic Fractures in Men (MrOs) Study. *Journal of the American Medical Directors Association*.

[36] Park S. W., Goodpaster B. H., Strotmeyer E. S. (2007). Accelerated loss of skeletal muscle strength in older adults with type 2 diabetes: the health, aging, and body composition study. *Diabetes Care*.

[37] Fung F. Y., Koh Y. L. E., Malhotra R. (2019). Prevalence of and factors associated with sarcopenia among multi-ethnic ambulatory older Asians with type 2 diabetes mellitus in a primary care setting. *BMC Geriatrics*.

[38] Fukuoka Y., Narita T., Fujita H. (2019). Importance of physical evaluation using skeletal muscle mass index and body fat percentage to prevent sarcopenia in elderly Japanese diabetes patients. *Journal of diabetes investigation*.

[39] Sazlina S.-G., Lee P. Y., Chan Y. M., MS A. H., Tan N. C. (2020). The prevalence and factors associated with sarcopenia among community living elderly with type 2 diabetes mellitus in primary care clinics in Malaysia. *PLoS One*.

[40] Batsis J. A., Villareal D. T. (2018). Sarcopenic obesity in older adults: aetiology, epidemiology and treatment strategies. *Nature Reviews Endocrinology*.

[41] Mesinovic J., McMillan L. B., Shore-Lorenti C., De Courten B., Ebeling P. R., Scott D. (2019). Metabolic syndrome and its associations with components of sarcopenia in overweight and obese older adults. *Journal of Clinical Medicine*.

[42] Su Y., Hirayama K. (2019). Sarcopenia prevalence and risk factors among Japanese community dwelling older adults living in a snow-covered city according to EWGSOP2. *Journal of Clinical Medicine*.

[43] Sousa-Santos A. R., Afonso C., Borges N. (2020). Sarcopenia, physical frailty, undernutrition and obesity cooccurrence among Portuguese community-dwelling older adults: results from Nutrition UP 65 cross-sectional study. *BMJ Open*.

[44] Liu X., Hao Q., Yue J. (2020). Sarcopenia, obesity and sarcopenia obesity in comparison: prevalence, metabolic profile, and key differences: results from WCHAT study. *The Journal of Nutrition, Health & Aging*.

[45] Kim J., Im J.-S., Choi C. H. (2018). The association between red blood cell distribution width and sarcopenia in US adults. *Scientific Reports*.

[46] Rolland Y., Czerwinski S., Van Kan G. A. (2008). Sarcopenia: its assessment, etiology, pathogenesis, consequences and future perspectives. *The Journal of Nutrition Health and Aging*.

[47] Aghili R., Malek M., Valojerdi A. E., Banazadeh Z., Najafi L., Khamseh M. E. (2014). Body composition in adults with newly diagnosed type 2 diabetes: effects of metformin. *Journal of Diabetes & Metabolic Disorders*.

